# Exploring the Association Between Clinical Characteristics and Etiopathogenesis of Tinnitus: A Cross-Sectional Study

**DOI:** 10.7759/cureus.70320

**Published:** 2024-09-27

**Authors:** Smriti Wadhwa, Shraddha Jain, Nimisha Patil, Harshil Dobariya, Vaibhavi Patil, Megha Kawale, Prasad T Deshmukh, Sagar S Gaurkar

**Affiliations:** 1 Otolaryngology and Head and Neck Surgery, Jawaharlal Nehru Medical College, Datta Meghe Institute of Higher Education and Research (Deemed to Be University), Wardha, IND

**Keywords:** cervicogenic tinnitus, non otologic tinnitus, otologic tinnitus, ringing sensation, tinnitus

## Abstract

Background

Tinnitus is the perception of internal noises like ringing or hissing, which can greatly affect quality of life. It involves increased neuronal synchrony and interactions with non-auditory brain structures. There’s no universal classification system due to its varied origins and mechanisms, and current protocols often overlook physiotherapy, especially for non-otologic causes such as Cervicogenic and Temporomandibular disorders. This study seeks to link clinical features with tinnitus characteristics to better identify underlying causes and improve diagnostic protocols.

Methods

A prospective observational cross-sectional study was conducted from June 2022 to March 2024 in a rural hospital in Central India, involving 75 patients with tinnitus. The study included comprehensive history, clinical examination, and physiotherapy assessments to categorize tinnitus into otologic, non-otologic, or central types. Each type was analyzed to correlate its characteristics with underlying causes. The findings aim to improve diagnostic protocols and deepen understanding of tinnitus origins.

Results

The otologic origin of tinnitus was found in 53 (70.7%) patients. Out of 16 (21.3%) patients in the non-otologic category, three (4%) of patients had a somatosensory origin, seven (9.3%) of patients were found to have a somatosensory cervicogenic origin of tinnitus, and six (8%) of patients had a central origin of tinnitus. The remaining six (8%) were found to have mixed origins.

Conclusion

Otologic tinnitus was the commonest cause of tinnitus, followed by cervicogenic tinnitus. Ours is probably the first study that classifies tinnitus based on otologic and non-otologic origin, with special emphasis on cervicogenic origin tinnitus. Further research could study tinnitus chronicity, causes, and treatments using objective measures and advanced diagnostics.

## Introduction

Tinnitus is the perception of ringing, hissing, clicking, or roaring sounds in the ears without external stimuli. It is not considered a separate medical condition but a symptom indicative of an underlying issue [1,2]. It can be either temporary or permanent. For some, it is a debilitating condition affecting daily life, while others adapt and manage well. Understanding its causes is crucial due to its potentially severe impact, with a global prevalence rate of about 10-15% of the population and an annual incidence rate estimated to be around 1-2% per year in the general population [3].

Tinnitus can stem from various causes, including inner, middle, or external ear pathologies like Meniere’s disease, otosclerosis, otitis media, or ototoxic medications. Noise-induced hearing loss (NIHL) is a common risk factor [4]. Research by Jafari has shown that the incidence of both tinnitus and hearing loss increases with age [5]. Non-otologic conditions include systemic causes, central, neuromuscular causes, or tumors. Central tinnitus is characterized by an abnormal sound that originates within the central nervous system. In certain cases, it may be associated with musculoskeletal issues rather than ear problems, termed "somatosensory tinnitus." These may also be linked to vertigo, hearing loss, or aural fullness, in addition to other clinical symptoms such as neck discomfort [6].

Pathophysiologically, tinnitus involves increased neuronal synchrony and abnormal brain activity beyond the cochlea. Research has expanded to include non-auditory structures and networks in the brain related to tinnitus [7]. The auditory system's link to the somatosensory system of the head and neck contains cervical tinnitus, a variant of somatosensory tinnitus [8].

Various classification schemes have been developed to differentiate between different types of tinnitus. These classifications include distinctions between pulsatile and non-pulsatile tinnitus, subjective and objective tinnitus, and primary and secondary tinnitus, as well as acute versus chronic cases. Pulsatile tinnitus is typically associated with vascular causes, while subjective tinnitus is only perceived by the individual affected, and objective tinnitus can be heard by both the patient and the examiner. The terms acute and chronic refer to the duration of tinnitus symptoms, whereas primary and secondary classifications indicate whether the tinnitus is related to sensorineural hearing loss or other factors [2,3].

The pathophysiology of tinnitus is complex and involves several potential mechanisms. Therefore, there is no universally accepted classification system for tinnitus due to the wide range of potential causes, and research is ongoing to understand its mechanisms and treatments. This study aims to classify the etiologies of tinnitus based on various clinical characteristics and diagnostic modalities into otologic and non-otologic tinnitus broadly. This research seeks to explore the relationship between clinical characteristics and associated symptoms with the causes of tinnitus to assist in identifying the source of tinnitus when multiple potential causes are present. This may be the first study aimed at diagnosing tinnitus origins with the potential to develop a diagnostic protocol for tinnitus of multiple etiologies.

## Materials and methods

The prospective observational study was conducted in the Department of Otorhinolaryngology at Jawaharlal Nehru Medical College, Datta Meghe Institute of Higher Education and Research (Deemed to Be University), Wardha, Central India, from June 2022 to March 2024 after approval from the Institutional Ethics Committee of Datta Meghe Institute of Medical Sciences (ref no. DMIMS(DU/IEC/2022/07)). The sample size was calculated using the Daniel formula: \[n = \frac{Z_{\alpha/2}^2 \cdot P (1-P)}{d^2}\]

where Z_α/2_​ is the level of significance at 5% (1.96) and P is the prevalence of tinnitus (10.1% or 0.101). A minimum of 75 patients were studied.

All the patients who presented with tinnitus in the otolaryngology department or were referred from other specialties like general medicine, neurology, neurosurgery, orthopedics, gynecology, and pediatrics were included in this study. Children below 10 years of age, post-operative tympanoplasty patients, and genetic diseases like Alport’s syndrome, Arnold Chiari syndrome, and Mondini dysplasia.

**Figure 1:**
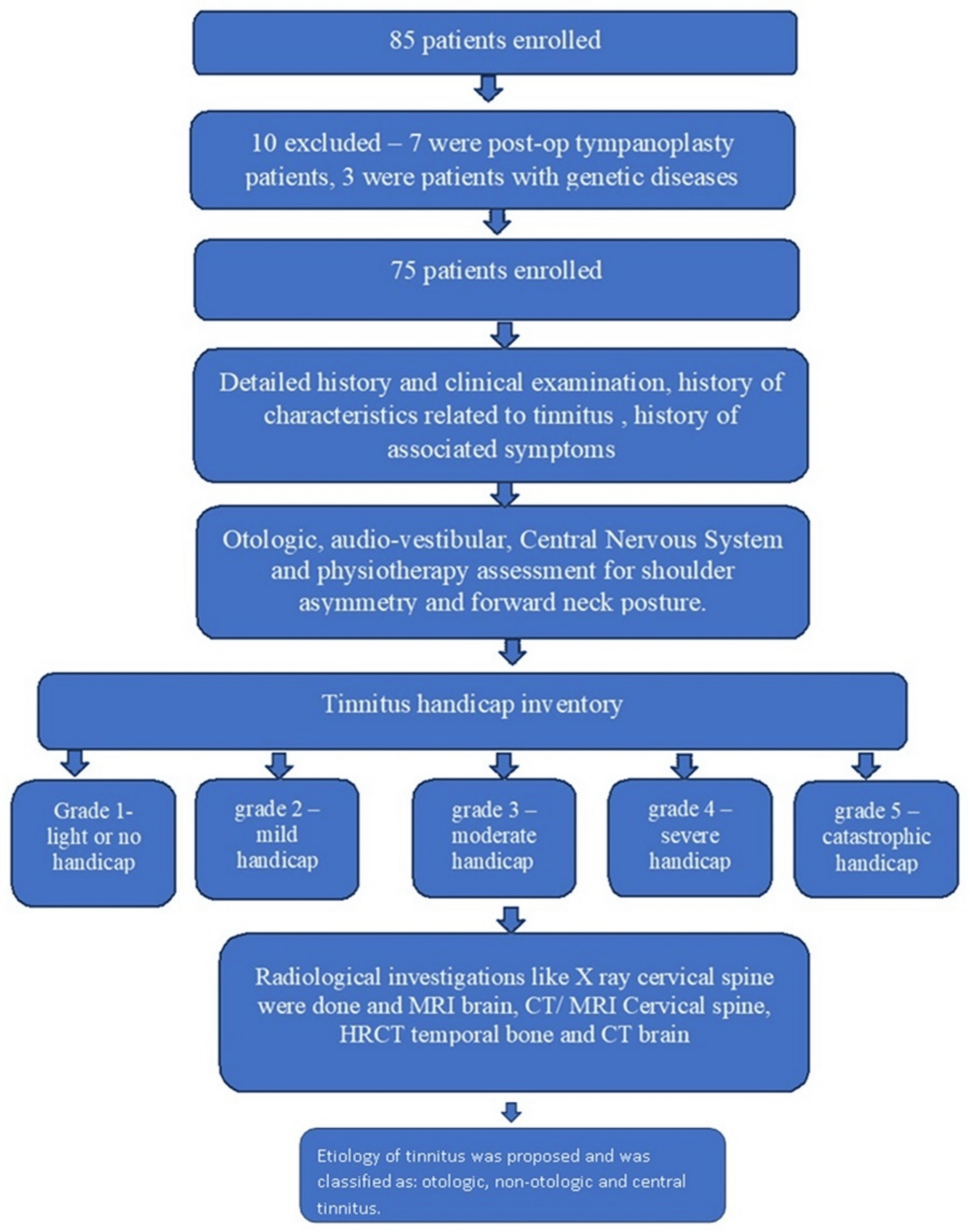
Methodology followed for the study HRCT: high resolution computed tomography; MRI: magnetic resonance imaging

The detailed methodology followed for this study is depicted in Figure 1.

In this study, 85 patients presenting with tinnitus at the Department of Otolaryngology or referred from other specialties (General Medicine, Neurology, Neurosurgery, Orthopedics, Gynecology, Pediatrics) were initially considered. Following the acquisition of written informed consent, patients were classified based on tinnitus origin into otologic, non-otologic, and mixed categories. Non-otologic tinnitus was subdivided into central, somatosensory, and cervicogenic types. Mixed categories were also further divided into mixed otologic with non-otologic central tinnitus, mixed otologic with non-otologic somatosensory tinnitus, and mixed otologic with non-otologic cervicogenic tinnitus.

After applying exclusion criteria, 75 patients were enrolled. A comprehensive clinical history was obtained by the otolaryngologist, focusing on tinnitus characteristics and associated symptoms. Clinical examinations included otologic, audiovestibular, general, systemic, and physiotherapy assessments. Audiological evaluations comprised Pure Tone Audiometry and Impedance Audiometry to assess hearing loss. Imaging studies included high-resolution computed tomography (HRCT) of the temporal bone to identify temporal bone-related causes of tinnitus and magnetic resonance imaging (MRI) of the brain for central tinnitus. X-rays of the cervical spine (anteroposterior and lateral views) were performed if cervicogenic tinnitus was suspected based on clinical history, with an MRI of the cervical spine conducted in specific cases where cervical spine pathology was considered.

Otologic tinnitus, originating from ear structures, was diagnosed based on associated ear symptoms and otoscopic findings. Non-otologic central tinnitus was identified through MRI brain imaging. Somatosensory tinnitus, linked to musculoskeletal disturbances, was diagnosed when patients exhibited related symptoms such as stress, dental issues, or temporomandibular joint disorders. Cervicogenic tinnitus was diagnosed based on symptoms like neck pain and restricted cervical motion, with cervical spine X-rays evaluated for structural abnormalities.

Primary outcome

To determine the proportion of patients diagnosed with different types of tinnitus (otologic, non-otologic, central, non-otologic Somatosensory, non-otologic cervicogenic, and mixed types) based on clinical history, otoscopic examination, and diagnostic investigations (audiometry, imaging studies like HRCT temporal bone, MRI brain, cervical X-rays).

To study various tinnitus characteristics (such as duration, severity, and laterality) and associated symptoms (neck pain, stress, unsteadiness, hearing loss, cervical spine abnormalities, temporomandibular joint disorders). This includes evaluating the diagnostic value of various clinical and audiological assessments in categorizing tinnitus types.

To find the association between tinnitus of different etiologies, its clinical characteristics, and associated symptoms.

Statistical analysis

Statistical analysis was performed using Epi Info TM version 7.2.5, a trademark of the Centers for Disease Control and Prevention. A descriptive statistical analysis was performed to calculate the mean and standard deviation. A test of proportion was used to find the standard normal deviation (Z), and the p-value less than 0.05 was considered statistically significant. Chi-squared test was used to explore the association between various etiopathologies and types of tinnitus.

## Results

This study was conducted on 75 patients. Their baseline characteristics were studied, where the youngest patient was 11 years old and the oldest patient was 88 years old with an average of 47 years and a standard deviation of 16.10. The most common age group affected was 41 to 55 years, with 26 (34.7%) patients. About 44 (58.7%) patients were found to be males, and 31 (41.3%) patients were females.

Other histories of importance included a history of noise exposure, hypertension, allergic rhinitis, depression, chronic kidney disease (CKD), chronic myeloid leukemia (CML), history of air travel, history of head injury, stress, and trauma to the ear in certain patients. Eight (10%) patients had a history of noise exposure.

About 49 (65.3%) patients had an associated symptom of reduced hearing, followed by giddiness in 24 (32%). Other symptoms included neck pain, ear fullness, ear discharge, earache, headache, nausea, and itching in the ear, as depicted in Table 1.

**Table 1:** Baseline characteristics of patients with tinnitus H/O: history of; CKD: chronic kidney disease; CML: chronic myelogenous leukemia

Basic details	Frequency (percentage)
Age group	
11 to 25 years	5 (6.7%)
26 to 40 years	22 (29.3%)
41 to 55 years	26 (34.7%)
56 to 70 years	17 (22.7%)
>70 years	5 (6.7%)
Gender	
Male	44 (58.7%)
Female	31 (41.3%)
Other significant history	
H/O noise exposure	8 (10.7%)
Hypertension	5 (6.7%)
Allergic rhinitis	4 (5.3%)
Depression	3 (4.0%)
CKD	1 (1.3%)
CML	1 (1.3%)
H/O air travel	2 (2.7%)
H/O head injury	2 (2.7%)
Stress	3 (4.0%)
Trauma to ear	3 (4.0%)
Associated symptoms	
Reduced hearing	49 (65.3%)
Giddiness	24 (32.0%)
Ear fullness	15 (20.0%)
Neck pain	14 (18.7%)
Ear discharge	6 (8.0%)
Earache	5 (6.7%)
Headache	5 (6.7%)
Nausea	2 (2.7%)
Itching in ear	1 (1.3%)

The patients were studied for various clinical characteristics of tinnitus like laterality, chronicity, type of tinnitus sounds, pitch of tinnitus, and tinnitus handicap based on the Tinnitus Handicap Inventory (THI) as depicted in Table 2. Forty-one (54.6%) patients complained of unilateral tinnitus, with 51 (68%) suffering from chronic tinnitus. Forty-seven (62.7%) patients had continuous tinnitus. Almost 45 (60%) patients were found to be having Grade 2 tinnitus, whereas no patients showed Grade 5 Catastrophic Handicap. Thirty (40%) patients had a low and moderate pitch of tinnitus each.

**Table 2:** Distribution of patients based on tinnitus characteristics THI: Tinnitus Handicap Inventory

Tinnitus	Frequency (percentage)
Laterality	
Bilateral	34 (45.3%)
Unilateral	41 (54.6%)
Chronicity	
Chronic	51 (68.0%)
Acute	24 (32.0%)
Type	
Whistling type	32 (42.7%)
Ringing type	14 (18.7%)
Flapping type	9 (12.0%)
Buzzing type	7 (9.3%)
Whooshing type	5 (6.7%)
Drum beat type	4 (5.3%)
Clicking type	2 (2.7%)
Pulsatile type	2 (2.7%)
Disability (according to THI)	
Grade 1	7 (9.3%)
Grade 2	45 (60.0%)
Grade 3	19 (25.3%)
Grade 4	4 (5.3%)
Pitch	
High	15 (20.0%)
Low	30 (40.0%)
Moderate	30 (40.0%)
Continuity	
Continuous	47 (62.7%)
Intermittent	28 (37.3%)

The pure tone audiometry findings of the patients with different etiologies of tinnitus are as in Table 3. The majority of 45 (43.7%) patients with tinnitus had sensorineural hearing loss.

**Table 3:** Distribution of patients based on type of hearing loss associated with tinnitus

Type of hearing loss	Frequency	Percentage
Conductive hearing loss	26	25.2%
Sensorineural hearing loss	45	43.7%
Mixed hearing loss	19	18.4%
Nil	13	12.6%

The various diagnoses have been described in Table 4. A maximum number of patients, 10 (13.3%) were found to have cervicogenic tinnitus, followed by eight (10.7%) with age-related hearing loss (ARHL), eight (10.7%) with NIHL, seven (9.3%) with chronic otitis media (COM), and seven (9.3%) with ETD. One in each of the 75 patients (1.3%) was also found to have tympanosclerosis, otomycosis, endolymphatic hydrops, sudden SNHL, temporomandibular joint disorder, aberrant vessels around the eighth nerve, vascular loops around the eighth nerve, meningioma, pineoblastoma, migranous tinnitus, vestibular schwannoma, and an infarct in the left corona radiata with left mastoiditis.

**Table 4:** Distribution of patients based on likely etiology of tinnitus ARHL: age-related hearing loss; NIHL: noise-induced hearing loss; COM: chronic otitis media; ETD: eustachian tube dysfunction; SOM: serous otitis media; AOM: acute otitis media Others (1.3% each) include tympanosclerosis, otomycosis, endolymphatic hydrops, sudden SNHL, temporomandibular joint disorder, aberrant vessels around the eighth nerve, vascular loops around the eighth nerve, meningioma, pineoblastoma, migranous tinnitus, vestibular schwannoma, infarct in left corona radiata with left mastoiditis.

Diagnosis	Frequency (percentage)
Cervicogenic tinnitus	10 (13.3%)
ARHL	8 (10.7%)
NIHL	8 (10.7%)
COM	7 (9.3%)
ETD	7 (9.3%)
SOM	6 (8.0%)
Otosclerosis	5 (6.7%)
Drug induced	4 (5.3%)
Cochlear otosclerosis	4 (5.3%)
Mixed otosclerosis	3 (4.0%)
Meniere's disease	3 (4.0%)
Head injury-induced tinnitus	3 (4.0%)
Stress-induced tinnitus	3 (4.0%)
AOM	2 (2.7%)
Barotrauma	2 (2.7%)
Labyrinthine fistula	2 (2.7%)
Trauma-induced labyrinthine concussion	2 (2.7%)
Others	12 (16%)

Based on the likely diagnosis of the origin of tinnitus, the etiologies were classified. Fifty-three (70.7%) patients had otologic tinnitus. Six (8.0%) patients had otologic central tinnitus. Three (4.0%) patients had non-otologic somatosensory tinnitus. Seven (9.3%) patients had non-otologic cervicogenic tinnitus as shown in Table 5.

**Table 5:** Division of various etiologies into three major categories - otologic, non-otologic, and mixed

Type of etiology		Frequency	Percentage
Otologic	Inner	22	41.5%
	Middle	21	39.6%
	Inner + middle	9	16.98%
	External	1	1.3%
	Total	53	70.7%
Non-otologic	Central	6	8.0%
	Somatosensory	3	4.0%
	Cervicogenic	7	9.3%
	Total	16	21.3%
Mixed		6	8.0%

The association between various etiopathology and the type of tinnitus sound showed a significant difference between the various groups in terms of the distribution type of tinnitus, as shown in Table 6. In the otologic group, 22 (41.5%) patients in the otologic group had whistling type tinnitus, followed by nine (17.0%) patients with ringing and flapping type of tinnitus. Others were buzzing, whooshing, and drumbeat types of tinnitus. In the otologic central group, three (50%) patients had the whistling type of tinnitus, two (33.3%) had a pulsatile type of tinnitus, and one (16.7%) had the pulsatile type of tinnitus. In the non-otologic somatosensory and cervicogenic group, patients were found to have whistling, ringing, and clicking types of tinnitus.

**Table 6:** The association between various etiopathologies and the type of tinnitus sound

Type of tinnitus sound	Etiology of tinnitus					χ^2^	p-value
	Otologic (53)	Non-otologic			Mixed (6)		
		Non-otologic central (6)	Non-otologic somatosensory (3)	Non-otologic cervicogenic (7)			
Whistling type	22 (41.5%)	3 (50.0%)	1 (33.3%)	3 (42.9%)	3 (42.9%)	73.6 46	0.0 02
Ringing type	9 (17.0%)	0	1 (33.3%)	3 (42.9%)	1 (33.3%)		
Flapping type	9 (17.0%)	0	0	0	0		
Buzzing type	7 (13.2%)	0	0	0	0		
Whooshing type	4 (7.5%)	0	0	0	1 (33.3%)		
Drum beat type	2 (3.8%)	1 (16.7%)	0	0	1 (33.3%)		
Clicking type	0	0	1 (33.3%)	1 (14.3%)	0		
Pulsatile type	0	2 (33.3%)	0	0	0		

The association between various otologic diagnoses, including inner, middle, and external, within both otologic and mixed etiologies and the type of tinnitus sound was done. A total of 34 patients each in inner and middle ear etiologies were found in otologic and mixed etiologies. It showed a significant difference in the middle ear and inner ear groups, as shown in Table 7. About 83.3% (five out of six) of patients with a whooshing type of tinnitus were found to have middle ear etiology. About 77.77% (seven out of nine) of patients who presented with a flapping type of tinnitus and 71.42% (five out of seven) with a buzzing type of tinnitus had middle ear etiology.

**Table 7:** The association between various otologic etiopathologies and the type of tinnitus sound

Type of otologic diagnosis (frequency)	Tinnitus: type of sound								χ^2^	p-value
	Whistle type	Ringing type	Flapping type	Buzzing type	Whooshing type	Drum beat type	Clicking type	Pulsatile type		
Middle ear (34)	14 (41.1%)	3 (8.8%)	7 (20.5%)	5 (14.7%)	5 (14.7%)	0	0	0	21.670	0.001
Inner ear (34)	16 (47.05%)	11 (32.35%)	1 (2.9%)	2 (5.88%)	1 (2.9%)	3 (8.82%)	0	0	17.602	0.008
External ear (1)	0	0	1 (100%)	0	0	0	0	0	7.432	0.387

Sixteen (47.05%) patients with inner ear etiology had the whistling type of tinnitus, and 11 (32.3%) had the ringing type of tinnitus. The drumbeat type of tinnitus was found exclusively in patients with inner ear etiology in our study.

There was no significant difference between the various groups in terms of disability of tinnitus and type of diagnosis, as shown in Table 8. Most patients were found to have grade 2 tinnitus, with 34 (64.2%) having otologic etiology, two (33.3%) with central etiology, two (66.7%) with somatosensory etiology, and four (57.1%) with cervicogenic etiology. Five (9.4%) patients with otologic etiology and one (33.3%) patients with somatosensory etiology of tinnitus showed grade 1 tinnitus. Grade 4 tinnitus was only found in otologic etiology in three (5.7%) patients and one (16.7%) patient with central etiology, whereas grade 3 was present in all etiologies.

**Table 8:** The association between disability according to Tinnitus Handicap Inventory (THI) and type of diagnosis

Tinnitus: disabling (according to THI)	Type of diagnosis					χ^2^	p-value
	Otologic (53)	Non-otologic			Mixed (6)		
		Central (6)	Somatosensory (3)	Cervicogenic (7)			
Grade 1	5 (9.4%)	0	1 (33.3%)	0	1 (16.6%)	17.1 13	0.389
Grade 2	34 (64.2%)	2 (33.3%)	2 (6.7%)	4 (57.1%)	3 (50%)		
Grade 3	11 (20.8%)	3 (50.0%)	0	3 (42.9%)	2 (33.3%)		
Grade 4	3 (5.7%)	1 (16.7%)	0	0	0		

Forty-two (79.2%) patients with otologic etiology had complaints of reduced hearing. Whereas, only two (33.3%) patients with central etiology complained of reduced hearing. Patients with non-otologic somatosensory etiology had the largest proportion of patients with normal hearing, as shown in Table 9. Three (50%) patients with central etiology had giddiness. One (33.3%) patient with somatosensory etiology had giddiness. Six (85.7%) patients with cervicogenic tinnitus had giddiness.

**Table 9:** The association between the type of diagnosis and the associated symptoms

Associated symptoms	Type of diagnosis					χ^2^	p-value
	Otologic (53)	Non-otologic			Mixed (6)		
		Central (6)	Somatosensory (3)	Cervicogenic (7)			
Reduced hearing	42 (79.2%)	2 (33.3%)	0	1 (14.3%)	4 (66.6%)	21.690	<0.001
Giddiness	11 (20.8%)	3 (50.0%)	1 (33.3%)	6(85.7%)	3 (60%)	21.044	<0.001
Ear fullness	15 (28.3%)	0	0	0	0	7.783	0.0053
Neck pain	0	2 (33.3%)	2 (66.7%)	6 (85.7%)	4 (66.6%)	51.790	<0.001
Ear discharge	6 (11.32%)	0	0	0	0	2.709	0.0998
Earache	4 (7.54%)	0	0	0	1 (16.6%)	2.173	0.1405
Headache	1 (1.88%)	2 (33.3%)	0	2 (28.57%)	0	14.844	<0.001
Nausea	1 (1.88%)	0	0	1 (14.3%)	0	4.175	0.0410
Itching in ear	1 (1.88%)	0	0	0	0	0.421	0.5164

Two (33.3%) patients in the central type of tinnitus had neck pain, two (66.7%) patients of the non-otologic somatosensory type had neck pain, and six (85.7%) with non-otologic cervicogenic etiology had neck pain.

Fifteen (28.3%) patients with otologic etiology had ear fullness. Two (33.3%) and two (28.57%) patients with central and cervicogenic etiology had headaches, respectively. There was no significant difference between the various groups in terms of ear discharge, earache, nausea, and itching in the ear.

## Discussion

In our research, out of 75 patients with tinnitus, 53 (70.7%) patients were found to have otologic etiology, whereas 22 (41.5%) and 21 (39.6%) had inner and middle ear etiology, respectively. Non-otologic etiology was found in 16 (21.3%), which included six (8%) with central etiology, three (4%) with somatosensory etiology, and seven (9.3%) with specifically cervicogenic etiology. Whereas, the rest six (8%) were found to have mixed etiologies.

The study included patients aged 11 to 88, with a mean age of 47.52 ± 16.10, which was consistent with the findings of Chen et al., in which the age was 45.7 ± 14.8. Aging is linked to hearing loss, and tinnitus often begins in middle age. Mean ages varied across groups: otologic (47.62), non-otologic central (31.16), non-otologic somatosensory (46.66), and non-otologic cervicogenic (52.00). Tinnitus causes were more common in those over 40, with cervicogenic dizziness associated with cervical spine issues and worsening with age [9,10]. However, no significant link between age and different etiologies was found (p = 0.27).

With regards to gender, our study had 44 (58.7%) males and 31 (41.3%) females, a 1.4:1 ratio. Chen et al. found 53.2% of males, and Hackenberg et al. reported 51.1% of males [9,11]. Arnold et al. found higher tinnitus prevalence in females [12]. Reporting and selection bias may exist as moderate tinnitus is often overlooked.

In terms of associated symptoms, 49 (65.3%) patients had reduced hearing, 24 (32.0%) experienced giddiness, and 14 (18.7%) had neck pain. According to Aryal et al., of the 100 individuals, 79% had normal hearing, 13% had mild reduced hearing, 6% had significantly reduced hearing, and 2% had full deafness [13]. Otologic patients mostly had reduced hearing, central patients had significant giddiness, and somatosensory patients frequently had neck pain [14,15]. Mixed cases showed the highest rates for both giddiness and reduced hearing.

In our study, eight (10.7%) patients had noise exposure. Dawes et al. linked tinnitus to neuroticism, noise exposure, hearing issues, low socio-economic status, and ototoxic drugs [16]. Others included five (6.7%) with hypertension, four (5.3%) with allergic rhinitis, three (4.0%) with depression, and one (1.3%) with CKD and CML each. Additionally, two (2.7%) had a history of air travel and head injury each, and three (4.0%) reported stress and ear trauma each.

Chronic tinnitus, often lasting over six months, significantly impacts quality of life. Our study showed 51 (68%) had chronic symptoms (>3 months) and 24 (32%) acute (<3 months). Wallhäusser-Franke E et al. found 68% of cases were permanent, with 73% starting suddenly [17]. Chronicity may result from underlying diseases, misdiagnosis, or undervaluing tinnitus [18].

Continuous and intermittent tinnitus are chronic, with transient tinnitus being a non-recurring acute episode [19]. In our study, 47 (62.7%) had continuous tinnitus, while 28 (37.3%) had intermittent. Nigerian and Brazilian studies reported varying proportions, with intermittent tinnitus being more common. Our higher continuous tinnitus rate may be linked to inner ear conditions [20]. Grade 2 was common in middle ear issues, grade 3 in inner ear and central causes, and grade 4 in both inner ear and central origins. Our study found 45 (60%) with grade 2 (mild) tinnitus and 19 (25.3%) with grade 3 (moderate). In the Beaver Dam Offspring Study (2005-2008) of over 3,000 adults aged 21-84, 10.6% reported tinnitus of at least moderate severity or difficulty falling asleep [21].

In our study, tinnitus pitch was evenly distributed: 15 (20%) high, 30 (40%) low, and 30 (40%) moderate, with no significant differences (p = 0.675). For research and sound treatments that depend on precise tinnitus pitch matching, the pitch of the tinnitus sound is a crucial feature. Otologic cases mainly had moderate pitch, central cases with moderate pitch had conditions like vascular loops, and somatosensory and cervicogenic cases mostly had low pitch [22,23].

The most common tinnitus sounds were whistling in 32 (42.7%) patients and ringing in 14 (18.7%). Other types included flapping, buzzing, whooshing, drum beat, clicking, and pulsatile tinnitus. Aryal et al. found ringing tinnitus most common in Indian patients (42%), followed by whistling (23%) and buzzing (14%) [13]. Whistling, ringing, and buzzing types were linked to various etiologies. Whistling was associated with inner ear issues, such as NIHL and Meniere's disease, and central causes like vestibular schwannoma. The ringing was most commonly linked to inner ear problems while buzzing, flapping, and whooshing were mainly tied to middle ear conditions. Pulsatile tinnitus was associated with central causes.

Five (83.3%) patients with whooshing tinnitus had middle ear pathology, specifically chronic otitis media, with one case of mastoiditis. Seven (77.8%) patients with a flapping type of tinnitus and five (71.4%) with buzzing tinnitus were linked to middle ear causes like eustachian tube dysfunction and severe otitis media. Pulsatile tinnitus was associated with central causes such as meningiomas and aberrant vessels around the eighth nerve. Clicking tinnitus was tied to somatosensory issues like temporomandibular joint disorder, while drum beat tinnitus was related to age-related hearing loss, cervicogenic issues, and central causes like Vascular Loops around the eighth nerve [24,25].

Ringing tinnitus is non-specific and linked to otologic, somatosensory, and cervicogenic causes. In inner ear otologic cases, 11 (32.35%) had a ringing type of tinnitus, associated with etiologies like ARHL and NIHL.

Whistling tinnitus, a non-specific type, was associated with various causes. In inner ear cases, 16 (47%) had whistling tinnitus, primarily due to NIHL and other conditions. In middle ear cases, the whistling type of tinnitus was linked to otosclerosis and chronic otitis media. It was also common in cervicogenic and central causes like vestibular schwannoma, migrainous tinnitus, and pineoblastoma [26,27].

Identifying the root cause of pulsatile tinnitus is essential for treatment and prognosis. Radiological imaging, particularly MRI for central and cervicogenic causes and HRCT for otologic causes, is crucial for diagnosing conditions such as tumors, vascular malformations, and anatomical anomalies [28,29]. Our study revealed various conditions, including vestibular schwannoma, vascular loops, pineoblastoma, and abnormalities in the cervical spine and temporal bone.

Mixed etiologies come with several difficulties. It might take a while and become increasingly difficult to pinpoint the exact source of tinnitus. For example, the coexistence of otologic hearing loss with cervical discomfort might complicate the clinical picture.

Strengths

This study is likely the first to classify tinnitus into otologic and non-otologic categories, including central, somatosensory, and cervicogenic causes. It also attempts to characterize tinnitus and correlate it with various etiologies, aiming to develop standard diagnostic and management protocols.

Limitations

It may be challenging to determine the precise cause of tinnitus in patients with mixed etiologies since they often present with a wide variety of symptoms. Also, subjective parameters of tinnitus were used for comparison. Our study was subject to the likelihood of biases like patient reporting bias and clinician bias, both of which can affect accuracy. It was avoided by providing patients with relatable sounds like the buzzing of an insect, the beating of a drum, the sound of air gushing through the ear, the sound of a whistle, the sound of paper flapping, and the sound of a ringing bell.

## Conclusions

The study provides an in-depth analysis of tinnitus by categorizing it into otologic, non-otologic, and mixed types. It finds that 70.7% of tinnitus cases are otologic, primarily linked to middle and inner ear issues. Non-otologic causes, including central and somatosensory origins, are less common. Tinnitus is more prevalent in older patients and males but with no significant links. Otologic cases were often found to be associated with reduced hearing, central cases with significant giddiness, and somatosensory cases frequently featured neck pain. A significant portion (68%) of patients had chronic tinnitus lasting over three months, with continuous tinnitus being notably prevalent. The THI grades show most patients have mild to moderate handicaps, with severe cases usually linked to inner ear or central issues. The most common tinnitus sound is whistling, followed by ringing and drumbeat, though non-specific. Different types of tinnitus suggest specific pathologies, like whooshing is linked to middle ear issues, pulsatile tinnitus to central causes, and clicking to temporomandibular joint or cervicogenic problems. Mixed etiologies complicate tinnitus diagnosis and often hide other conditions. Comprehensive assessment and imaging, like MRI and HRCT, are crucial. The study stresses the need for a detailed approach in such cases.

Key recommendations and areas of further research emphasize the importance of studying tinnitus chronicity and burden in relation to patient factors, disease pathophysiology, and treatment gaps. It is important to use objective parameters to link tinnitus to different causes. Cohort studies should be conducted to develop targeted treatments, and the exploration of myofascial tightness in somatosensory tinnitus is suggested. Additionally, advanced diagnostics should be applied to pinpoint causes, and larger sample sizes should be used in research.
